# Determination and Application of Pont's Index in Turkish Population

**DOI:** 10.1100/2012/494623

**Published:** 2012-05-01

**Authors:** Ahmet Arif Celebi, Enes Tan, Ibrahim Erhan Gelgor

**Affiliations:** Department of Orthodontics, Faculty of Dentistry, Kirikkale University, Merkez, 71200 Kirikkale, Turkey

## Abstract

Disharmony between tooth size and dental arch size induces orthodontic problems. So, dental indices were identified by various authors. One of these is Pont who determined a method of prediction of the ideal dental arch width which has become known as Pont's Index. The purpose of this study was to assess the applicability of Pont's Index to a Turkish population and to compare the results with those obtained from studies of different ethnic subjects. The sample comprised 64 male subjects and 78 female subjects with age range from 14 to 15 years. Measurements were obtained directly from plaster casts; they included mesiodistal crown diameters of the four maxillary incisors, as well as interpremolar and intermolar maxillary arch widths as specified by Pont. Correlation coefficients determined between the measured arch width values and those calculated according to Pont's Index were low in all cases, with *r* values ranging from 0.02 to 0.36. It was concluded that Pont's Index should not be used to predetermine ideal arch width values in Turkish individuals.

## 1. Introduction

Transverse or vertical arch male relationships such as crowding and local irregularities are common causes of Class I malocclusions and are handled usually by extraction or nonextraction treatment in the permanent dentition [1].

Various diagnostic indices have been proposed in clinical orthodontics which helps to predict dental arch growth and assist with treatment planning. In orthodontic treatment, a wealth of information obtained from dental casts plays a significant role in diagnosis, treatment planning and evaluation [2]. One of the dental cast evaluations was described by Pont who found that the ideal arch width necessary to accommodate the dentition and relieve crowding can be determined by assuming a constant relationship between the sum of the mesiodistal widths of the permanent maxillary incisors and the interpremolar or intermolar arch widths [3]. His indices were determined by dividing the sum of the incisal widths (SIW) × 100 by the respective arch widths. The interpremolar arch width (IPW) was taken from the first premolar of the left side to the right side at the distal end of its occlusal groove. The molar arch width (IMW) was taken from the maxillary left first permanent molar to the same of the right at its mesial pit on the occlusal surface. Based on an ideal occlusion sample, the values of 80 and 64 were calculated by him for the premolar index and the molar index, respectively. He also prepared a prediction table from which ideal first premolar arch width and the ideal intermolar width could be read directly after finding the mesiodistal diameters of the maxillary incisor teeth.

(1) $$\begin{array}{cc} & \text{Interpremolar}\;\text{arch}\;\text{width}\;\left(\text{IPW}\right)\\ & \;\;=\frac{\text{Sum}\;\text{of}\;\text{the}\;\text{incisal}\;\text{widths}\;\left(\text{SIW}\right)}{0.80},\\ & \text{Intermolar}\;\text{arch}\;\text{width}\;\left(\text{IMW}\right)\\ & \;\;=\frac{\text{Sum}\;\text{of}\;\text{the}\;\text{incisal}\;\text{widths}\;\left(\text{SIW}\right)}{0.64}. \end{array}$$

Pont obtained his data from an ill-defined French population and did not indicate how many subjects were included in his sample. Nevertheless, he apparently was aware of possible differences between ethnic groups and suggested that the reliability of his index should be tested in other populations. Some investigators are supporting its use to predict arch widths [4, 5], while others believe that Pont's Index is not reliable and should not be used for clinical purposes [6–9].

The present study was initiated to provide estimates of Pont's Index in selected samples Turkish subjects and also to enable comparisons to be made with previously published reports from other ethnic groups.

## 2. Methods

A sample of 142 subjects (64 male subjects and 78 female subjects with age range of 14-15; mean age 14.24 ± 0.64 years) was randomly selected from a population that attended the Dental Faculty of Kirikkale (Kirikkale city) in the centre of Anatolia, Turkey. The sample was derived from general dental health control demanded subjects not seeking the orthodontic treatment. The examinations were carried out in the oral diagnosis clinics. Family origin, registered in order to determine the Turkish racial composition of the sample, was found to be representative of Anatolian ancestry from the central part of the country. It was identified that the mothers and fathers of all the individuals were Turkish.

The following criteria were used for selection of the sample:

1. angle class I occlusal relationship with normal overbite and overjet (overbite < 4 mm, overjet < 3 mm),
2. well-aligned upper and lower dental arches,
3. normal growth and development pattern,
4. no history of previous orthodontic or prosthodontic treatment,
5. full complement of teeth from second molar to second molar in both arches,
6. no missing teeth, no supernumerary teeth.

The sample size was calculated as 142 patients per group, based on a significance level of 0.05, a power of 80%.

Alginate impressions were taken for individuals. The impressions were poured on the same day with hard dental stone. Measurements of all study models were done using a digital caliper with sharpened beaks (accuracy of 0.01 mm). Systematic and random errors were minimized by rigidly standardizing experimental equipment and procedures. To determine the errors associated with cast measurements, 25 cast model were selected. Their measurements were repeated 2 weeks after the first measurement and made by the same observer. The landmarks used for measurements were as follows:

- maxillary interpremolar width: distal pits of the maxillary first premolars,
- maxillary intermolar width: central fossae of the maxillary first molars.

Mean differences between replicated measurements representing tooth size and arch dimensions were not significantly different from zero. The mean errors calculated using Dahlberg's formula [10] ranged from 0.06 to 0.24 mm for tooth size measurements and from 0.22 to 0.31 mm for arch width measurements. The coefficients of reliability calculated as recommended by Houston [11] ranged from 93 to 99 per cent for tooth width measurements and from 95 to 98 per cent for arch width measurements. These findings indicated that experimental errors were generally small and unlikely to bias the results.

### 2.1. Statistical Analysis

All statistical analyses were performed using the Statistical Package for Social Sciences (Windows, version 16.0; SPSS Inc., Chicago, IL, USA). Average values, standard deviations, and coefficients of variation were calculated for males and females separately. Incisor and arch widths were recorded for each subject to the nearest 0.01 mm according to Pont's formulae, arch widths were calculated for each subject and the correlation coefficients were calculated between the measured and the calculated arch width values. The independent samples *t*-test was used to detect whether there was a significant difference in tooth and/or arch width values for males and females (Table 1).

## 3. Results

Even though males had significantly bigger values for incisor widths, there was no significant difference between incisor tooth size in males and females. Also, nearly all of the arch width measurements in males did not differ significantly from females (Table 1).

Coefficients correlation was ascertained between combined maxillary incisor widths to premolar arch width, and to molar arch width and the corresponding values that were calculated according to Pont's Index were low in all cases for males and females, with *r* values ranging from 0.02 to 0.36 (Table 2).

**Figure 1:**
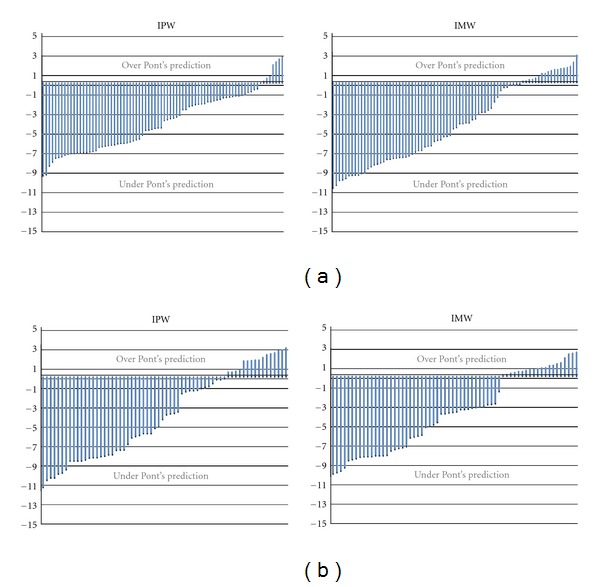
(a) Differences between measured and predicted arch width values for females in millimetres. (b) Differences between measured and predicted arch width values for males in millimetres.

The differences between measured and calculated arch width values that were calculated for females and males are presented in Table 3 and Figure 1.

## 4. Discussion

The importance of tooth size discrepancies in orthodontic diagnosis has been widely reported in the literature and accepted by the orthodontic community because the relationship between the upper and lower anterior and posterior dentitions is related to orthodontic finishing excellence [12]. Because of this, many indices and methods have been suggested to guide clinicians in predicting the ideal arch width [13–15]. One of these was described by Pont [3] who obtained his data from an ill-defined French population and did not indicate how many subjects were included in his sample. However, he apparently was aware of possible differences between ethnic groups and suggested that the reliability of his index should be tested in other populations. Genetic influences have been considered important in the determination of tooth dimensions, and the first reports were related to clinical observations within families. Studies on twins, however, helped in understanding the genetic contribution of tooth size in that a greater tooth size correlation was found in monozygotic twins [16, 17]. Tooth size differences exist among various ethnic groups, and it is reported that individuals of Black ethnic backgrounds have larger teeth than Caucasians. Studies including Hispanic populations reported significant differences in relation to Caucasians but tooth size similarities to African Americans. The Brazilian population, like the Hispanic population, is composed of a mixture of African and European descendents [18–20].

In this study, teenagers were chosen to minimize the alteration of the mesiodistal tooth dimensions because of factors such as attrition, restoration, or caries. This is the first study that aimed to determine Pont's Index on Turkish population.

The results from the present study showed no statistically significant differences in the maxillary incisor widths among the genders (Table 1). This disagrees with the findings of Karaman [21] who determined that Turkish male teeth have larger widths than female teeth. The difference was statistically significant. In other study, Al-Omari et al. [22] compared the dimensions of teeth in Jordanian population and found no significant differences in maxillary incisor widths among the genders.

The correlations between measured arch width in premolar and molar area and its corresponding calculated arch width according to Pont's formulae were low (Table 2). So, we may say Pont's Index cannot provide reliable predictions for individual orthodontic treatment planning. These findings are in agreement with those reported by other investigators [6, 7, 23]. Some persons were “over Pont's prediction,” which means that their observed arch widths were larger than those predicted by Pont's Index. On the other hand, some persons were “under Pont's prediction” indicating that their observed arch widths were less than expected according to Pont's Index (Figures 1(a) and 1(b)). Since only 12.5% of males, 7.7% of females (for interpremolar widths), and 18.8% of males, 20.5 females (for intermolar widths) arch widths demonstrated no differences between −1 mm and +1 mm of Pont's Index estimates. Therefore, these differences show that Pont's Index tends to overestimate the arch width required to relieve crowding (Figures 1(a) and 1(b)) [24]. Al-Omari et al. and Dalidjan et al. found these values to be similarly low for the populations in which they applied the index.

It has been suggested that the generalized use of the Pont index might not be valid for other populations [6, 21, 25]. On the other hand, studies suggesting that Pont index is applicable to other populations are present as well [4, 5, 7]. Based on these different studies results, Pont's Index was originally founded on the mean value of an unspecified French population; individual variations and population differences were not covered.

## 5. Conclusion

After examining diagnostic dental casts taken during the mixed dentition stage in 142 untreated subjects, we reached the following conclusion.

Pont Index is not reliable for predetermination of ideal arch width values for Turkish males and females.

## Figures and Tables

**Table 1:** Tooth size and dental arch width (in mm) for males and females described in terms of arithmetic means (AVG), standard deviation (SD), and coefficient of variation (CV).

	Variable	Males (*n* = 64)			Female (*n* = 78)			Statistical comparison
		AVG	SD	CV	AVG	SD	CV	*P* value
Tooth widths	12	7.12	0.58	8.14	6.98	0.66	9.45	0.45
	11	8.9	0.55	6.17	8.86	0.47	5.3	0.81
	21	8.9	0.55	6.17	8.86	0.47	5.3	0.81
	22	7.16	0.57	7.96	6.97	0.66	9.46	0.32

Arch width	IPW	35.95	4.33	12.04	35.56	2.62	7.36	0.68
	IMW	46.25	3.92	8.47	45.18	2.77	6.13	0.26

*Significant differences between males and females at *P* < 0.05.

**Table 2:** Correlation coefficient (*r*) and coefficients of determination (*r* ^2^) between measured and calculated arch width values according to Pont's formulae.

	Males (*n* = 64)			Females (*n* = 78)		
Arch widths	*r*	*r* ^2^	*P*	*r*	*r* ^2^	*P*
Interpremolar	0.07	0.006	0.77	0.24	0.06	0.13
Intermolar	0.36	0.13	0.16	0.02	0.001	0.86

**Table 3:** Percentage of individuals having an observed arch width values under, over, and ±1 mm around Pont's prediction.

		Under Pont's prediction	Over Pont's prediction	Pont's prediction ±1 mm
IPW	Males (*n* = 64)	68.8	18.7	12.5
	Females (*n* = 78)	84.6	7.7	7.7
IMW	Males (*n* = 64)	68.8	12.4	18.8
	Females (*n* = 78)	66.7	12.8	20.5
